# Mck1 kinase is a new player in the DNA damage checkpoint pathway

**DOI:** 10.1371/journal.pgen.1008372

**Published:** 2019-10-31

**Authors:** Nerea Sanvisens Delgado, David P. Toczyski

**Affiliations:** UCSF Helen Diller Comprehensive Cancer Center, Univerisity of Califorinia, San Francisco, California, United States of America; The University of North Carolina at Chapel Hill, UNITED STATES

The DNA damage checkpoint is a signal transduction cascade with three layers of kinases. Checkpoint kinases are conventionally defined as kinases that are activated by DNA damage—either directly or by upstream kinases—and phosphorylate targets that preserve genome stability. In the presence of damaged DNA, the sensor kinases (ATM/ATR in mammals and Tel1/Mec1 in budding yeast) become active and phosphorylate the effector kinases (CHK1 and CHK2 in mammals and Chk1 and Rad53 in budding yeast [1]). Rad53, in turn, activates another checkpoint kinase, Dun1 [2]. This hierarchy is likely to provide both amplification and specialization, as the substrates of each set of kinases are selectively enriched for proteins in particular areas of biology. ATM and ATR are localized to DNA breaks, and their substrates are enriched for chromatin components and repair proteins that are similarly localized (e.g., H2AX and Slx4) [3–7]. While Rad53, Chk1, and their homologues target some proteins in this category, they primarily act on a large number of substrates that are not directly adjacent to sites of DNA damage, including cell cycle regulators, such as Sld3 [8, 9] and Pds1 [10]. By contrast, Dun1’s only known substrates are involved in the regulation of ribonucleotide levels [11]. Here, Liu and colleagues [12] show that the GSK3-related kinase Mck1 is directly activated by Rad53 and, like Dun1, regulates ribonucleotide biosynthesis, suggesting it too is a checkpoint kinase.

All eukaryotic organisms require an adequate concentration of deoxyribonucleoside triphosphates (dNTPs) in order to ensure accurate DNA replication and repair and to maintain genomic stability. The rate-limiting step in dNTP synthesis is catalyzed by ribonucleotide reductase (RNR), an essential heterotetrameric enzyme that mediates the reduction of ribonucleotides (rNTPs) into deoxyribonucleotides (dNTPs). In the budding yeast *Saccharomyces cerevisiae*, the large R1 subunit is composed of an Rnr1 homodimer (or Rnr1-Rnr3 heterodimer), whereas the active small R2 subunit is formed by an Rnr2-Rnr4 heterodimer [13]. The activity of RNR is tightly regulated by the cell cycle and environmental cues, which is critical since an unbalanced supply of dNTPs dramatically increases the mutation rate. Once Dun1 becomes activated, it enhances RNR activity by multiple mechanisms. First, in response to DNA damage and replication stress, Dun1 phosphorylates the Crt1 repressor. This causes it to be lost from *RNR* promoters, leading to an increase in transcription of *RNR2*, *RNR3*, and *RNR4*. However, induction of *RNR* genes upon genotoxic stress is not completely dependent upon the Dun1 kinase. In *dun1Δ* mutants, *RNR* genes continue to be significantly induced in response to DNA damage [14]. *DUN1-*independent *RNR1* induction upon DNA damage is also Crt1-independent. This is mediated by Rad53 activation of Ixr1, a DNA-binding protein that interacts with the *RNR1* promoter and activates *RNR1* transcription [15]. In addition, the yeast Rnr1 inhibitor Sml1 undergoes a *DUN1*-dependent phosphorylation that leads to Sml1 degradation [16–18]. A third mechanism regulates the subcellular distribution of the RNR subunits in yeast. Under normal conditions, the large subunit R1 is predominantly localized to the cytoplasm, whereas the small subunit R2 localizes to the nucleus, except during S phase [19]. The nuclear localization of the small subunit R2 is achieved by a dual mechanism. Under normal growth conditions, the nuclear WD40 protein Wtm1 binds to Rnr2-Rnr4 and anchors the complex to the nucleus, limiting its export, whereas Dif1 facilitates the nuclear import of the Rnr2-Rnr4 heterodimer by directly interacting with the complex. Dun1 disrupts the association between Rnr2-Rnr4 and Wtm1 in the nucleus, leading to release of Rnr2-Rnr4 into the cytoplasm, where it presumably assembles with the large subunit R1, resulting in an active RNR complex. Simultaneously, Dif1 is phosphorylated by Dun1 and degraded, thereby diminishing nuclear import [20, 21].

In this study, Liu and colleagues [12] show that the highly conserved GSK3-related Mck1 kinase is a downstream target of Rad53 and functions in the Dun1-independent RNR activation pathway. The authors suggest that Mck1 and Dun1 kinases cooperate in a nonredundant manner to provide cells with a multilayer response system to deal with various degrees of replicative stress. Using a synthetic genetic screen, Liu and colleagues [12] find that deletion of *MCK1* and *DUN1* (but not other *GSK3* paralogs) displays a synergistic sensitivity to replication stress, reminiscent of *mec1Δ* or *rad53Δ*. They show that, like Dun1, Mck1 is phosphorylated by Rad53, and genetic experiments suggest that this phosphorylation is activating. Mck1 appears to act on both *CRT1* itself and a *CRT1*-independent pathway. The authors demonstrated that Crt1 phosphorylation is significantly compromised in an *MCK1* deletion, accompanied with dissociation of Crt1 from the *RNR* promoter, resulting in induction of RNR genes. This phosphorylation is only partially redundant with Crt1 phosphorylation by Dun1. In addition, the authors demonstrate that Mck1 represses the transcription of *HUG1* in a Crt1-independent way. This observation is reminiscent of previous work showing that Hug1 acts to fine-tune RNR activity [22]. According to the authors’ model, when higher levels of RNR activity are required after cells suffer a more severe condition, Mck1 will inhibit the induction of *HUG1* in a Crt1-independent manner (Fig 1).

**Fig 1:**
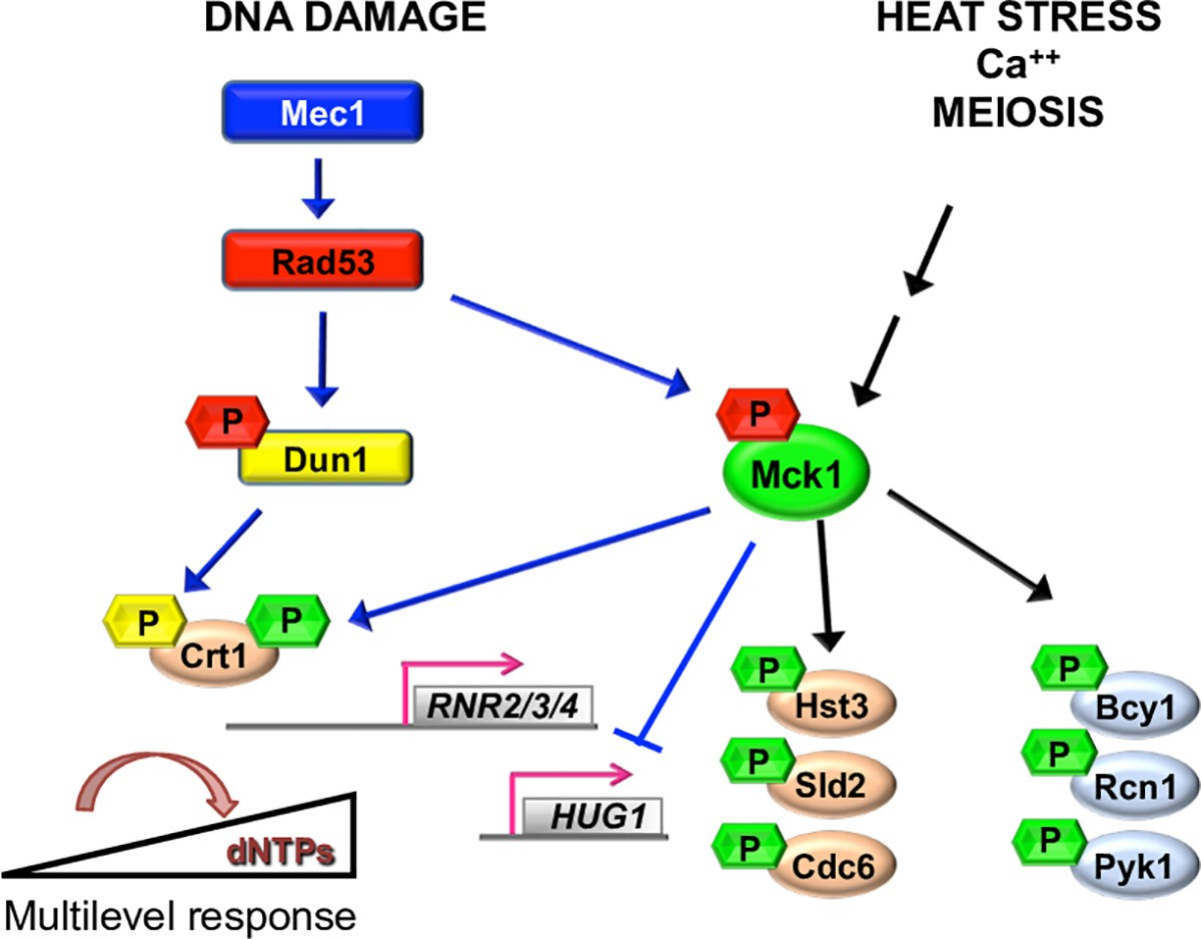
Multiple roles of Mck1 in response to stress. Upon DNA damage, Mck1, together with Dun1, antagonizes the repressor function of Crt1 via phosphorylation, which allows the derepression of *RNR2/3/4* transcription. Meanwhile, Mck1 inhibits the expression of the RNR inhibitor Hug1 in a Crt1-independent manner. This mechanism allows the cell to maintain appropriate dNTPs levels according to the degree of stress. Yeast Mck1 has been shown to phosphorylate the cell cycle regulators Cdc6, Hst3, and Sld2, the calcineurin regulator Rcn1 to stimulate calcineurin signaling, Pyruvate Kinase 1 (Pyk1), and the PKA regulatory subunit Bcy1 and to have roles in meiosis, sporulation, and heat stress resistance.

Liu and colleagues’ discovery that Mck1 is directly activated by Rad53 is particularly interesting given previous connections between Mck1 and the DNA damage response. In response to DNA damage, Mck1 phosphorylates the PKA regulatory subunit Bcy1, restraining anaphase [23]. Furthermore, Mck1 and Rad53 activities are required to promote Hst3 turnover by the ubiquitin ligase SCF^Cdc4^ to maintain genome stability in response to replication stress [24]. Interestingly, Mck1 also seems to be important to ensure proper DNA replication, prevent DNA damage, and maintain genome integrity by promoting Cdc6 degradation after DNA damage [25]. While each of the above mentioned studies suggested that Mck1 activity was important for the response to DNA damage, they did not show that it was directly activated by DNA damage, thus fulfilling the definition of a checkpoint kinase. Several other yeast kinases, such as casein kinase—and even cyclin-dependent kinase—clearly phosphorylate proteins important for the damage response; however, they are not directly activated by the checkpoint pathway [26–29]. Thus, unlike Mck1, they may be important for the damage response but are not strictly DNA damage checkpoint kinases.

Like Mck1, several mammalian kinases with previously characterized roles in other pathways also appear to moonlight in the DNA damage response. Vertebrate GSK-3 phosphorylates the oncogenic transcription factor c-Myc after ultraviolet light, targeting it for ubiquitination by SCF^Fbw7^ [30, 31]. In addition, several vertebrate MAP kinases (MAPKs) have links with the damage response. There are three subgroups of mammalian MAPKs: extracellular signal regulated kinases (ERKs), stress-activated protein kinase/jun N-terminal kinase (SAPK/JNK), and p38 MAPKs. Both MK2 and JNK can be activated in an ATM-dependent manner in response to particular genotoxic stresses [32–35]. These kinase pathways are involved in the cellular response to environmental stresses but can also be modulated during apoptosis, transformation, development, immune activation, and inflammation in an ATM-independent manner. Although these kinases are not entirely dedicated to the DNA damage response, there is some evidence that other signals may input into the traditional checkpoint kinases as well. For example, ATM is thought to be activated by oxidation stress unrelated to DNA damage [36, 37]. One question that remains from these studies, however, is whether the substrates of these noncanonical checkpoint kinases vary depending upon the way in which they have been activated. In addition to the cell cycle regulators Cdc6, Hst3, and Sld2; yeast Mck1 has been shown to phosphorylate the calcineurin regulator Rcn1 [38] and Pyruvate Kinase 1 [39] and to have roles in meiosis and sporulation [40]. Mck1’s targeting of Bcy1 has been shown to be regulated by heat shock [41] as well as DNA damage [23]. The fact that multiple inputs lead to activation of Mck1 leaves open the question of whether disparate signals activating it lead to phosphorylation of only a subset of its substrates, as has been characterized for other yeast MAP kinases [42], or whether one signal, such as DNA damage or heat shock, impinges upon all Mck1-regulated pathways.

## References

[1] Ciccia A, Elledge SJ. The DNA Damage Response: Making It Safe to Play with Knives. Molecular Cell. 2010; 10.1016/j.molcel.2010.09.019 20965415 PMC2988877

[2] Chen SH, Smolka MB, Zhou H. Mechanism of Dun1 activation by Rad53 phosphorylation in Saccharomyces cerevisiae. J Biol Chem. 2007; 10.1074/jbc.M609322200 17114794 PMC2811688

[3] Rogakou EP, Pilch DR, Orr AH, Ivanova VS, Bonner WM. DNA double-stranded breaks induce histone H2AX phosphorylation on serine 139. J Biol Chem. 1998; 10.1074/jbc.273.10.5858 9488723

[4] Ward IM, Chen J. Histone H2AX Is Phosphorylated in an ATR-dependent Manner in Response to Replicational Stress. J Biol Chem. 2001; 10.1074/jbc.C100569200 11673449

[5] Ohouo PY, Bastos de Oliveira FM, Almeida BS, Smolka MB. DNA damage signaling recruits the Rtt107-Slx4 scaffolds via Dpb11 to Mediate replication stress response. Mol Cell. 2010; 10.1016/j.molcel.2010.06.019 20670896

[6] Cussiol JR, Dibitetto D, Pellicioli A, Smolka MB. Slx4 scaffolding in homologous recombination and checkpoint control: lessons from yeast. Chromosoma. 2017; 10.1007/s00412-016-0600-y 27165041 PMC5104683

[7] Dibitetto D, Ferrari M, Rawal CC, Balint A, Kim T, Zhang Z et al Slx4 and Rtt107 control checkpoint signalling and DNA resection at double-strand breaks. Nucleic Acids Res. 2016; 10.1093/nar/gkv1080 26490958 PMC4737138

[8] Lopez-Mosqueda J, Maas NL, Jonsson ZO, Defazio-Eli LG, Wohlschlegel J, Toczyski DP. Damage-induced phosphorylation of Sld3 is important to block late origin firing. Nature. 2010; 10.1038/nature09377 20865002 PMC3393088

[9] Zegerman P, Diffley JFX. Checkpoint-dependent inhibition of DNA replication initiation by Sld3 and Dbf4 phosphorylation. Nature. 2010; 10.1038/nature09373 20835227 PMC2948544

[10] Sanchez Y, Bachant J, Wang H, Hu F, Liu D, Tetzlaff M et al Control of the DNA damage checkpoint by Chk1 and Rad53 protein kinases through distinct mechanisms. Science. 1999; 10.1126/science.286.5442.1166 10550056

[11] Zhou Z, Elledge SJ. DUN1 encodes a protein kinase that controls the DNA damage response in yeast. Cell. 1993; 10.1016/0092-8674(93)90321-G 8261511

[12] Li Xiaoli, Jin Xuejiao, Sharma Sushma, Liu Xiaojing, Zhang Jiaxin, Niu Yanling et al Mck1 defines a key S-phase checkpoint effector in response to various degrees of replication threats. PLoS Genet. 2019 10.1371/journal.pgen.1008136 31381575 PMC6695201

[13] Lee YD, Elledge SJ. Control of ribonucleotide reductase localization through an anchoring mechanism involving Wtm1. Genes Dev. 2006; 10.1101/gad.1380506 16452505 PMC1361704

[14] Huang M, Zhou Z, Elledge SJ. The DNA replication and damage checkpoint pathways induce transcription by inhibition of the Crt1 repressor. Cell. 1998; 10.1016/S0092-8674(00)81601-3 9741624

[15] Tsaponina O, Barsoum E, Åström SU, Chabes A. Ixr1 is required for the expression of the ribonucleotide reductase Rnr1 and maintenance of dNTP pools. PLoS Genet. 2011; 10.1371/journal.pgen.1002061 21573136 PMC3088718

[16] Chabes A, Domkin V, Thelander L. Yeast Sml1, a protein inhibitor of ribonucleotide reductase. J Biol Chem. 1999; 10.1074/jbc.274.51.36679 10593972

[17] Zhao X, Muller EGD, Rothstein R. A suppressor of two essential checkpoint genes identifies a novel protein that negatively affects dNTP pools. Mol Cell. 1998; 10.1016/S1097-2765(00)80277-4 9774971

[18] Zhao X, Chabes A, Domkin V, Thelander L, Rothstein R. The ribonucleotide reductase inhibitor Sml1 is a new target of the Mec1/Rad53 kinase cascade during growth and in response to DNA damage. EMBO J. 2001; 10.1093/emboj/20.13.3544 11432841 PMC125510

[19] Yao R, Zhang Z, An X, Bucci B, Perlstein DL, Stubbe J, et al Subcellular localization of yeast ribonucleotide reductase regulated by the DNA replication and damage checkpoint pathways. Proc Natl Acad Sci. 2003; 10.1073/pnas.1131932100 12732713 PMC164498

[20] Lee YD, Wang J, Stubbe JA, Elledge SJ. Dif1 Is a DNA-Damage-Regulated Facilitator of Nuclear Import for Ribonucleotide Reductase. Mol Cell. 2008; 10.1016/j.molcel.2008.08.018 18851834 PMC3245869

[21] Wu X, Huang M. Dif1 Controls Subcellular Localization of Ribonucleotide Reductase by Mediating Nuclear Import of the R2 Subunit. Mol Cell Biol. 2008; 10.1128/mcb.01388-08 18838542 PMC2593381

[22] Meurisse J, Bacquin A, Richet N, Charbonnier JB, Ochsenbein F, Peyroche A. Hug1 is an intrinsically disordered protein that inhibits ribonucleotide reductase activity by directly binding Rnr2 subunit. Nucleic Acids Res. 2014; 10.1093/nar/gku1095 25378334 PMC4245953

[23] Searle JS, Wood MD, Kaur M, Tobin D V., Sanchez Y. Proteins in the Nutrient-Sensing and DNA damage checkpoint pathways cooperate to restrain mitotic progression following DNA damage. PLoS Genet. 2011; 10.1371/journal.pgen.1002176 21779180 PMC3136438

[24] Edenberg ER, Vashisht AA, Topacio BR, Wohlschlegel JA, Toczyski DP. Hst3 is turned over by a replication stress-responsive SCFCdc4 phospho-degron. Proc Natl Acad Sci. 2014; 10.1073/pnas.1315325111 24715726 PMC4000829

[25] Al-Zain A, Schroeder L, Sheglov A, Ikui AE. Cdc6 degradation requires phosphodegron created by GSK-3 and Cdk1 for SCF Cdc4 recognition in Saccharomyces cerevisiae. Mol Biol Cell. 2015; 10.1091/mbc.e14-07-1213 25995377 PMC4501359

[26] Toczyski DP, Galgoczy DJ, Hartwell LH. CDC5 and CKII control adaptation to the yeast DNA damage checkpoint. Cell. 1997; 10.1016/S0092-8674(00)80375-X 9323137

[27] Bonilla CY, Melo JA, Toczyski DP. Colocalization of Sensors Is Sufficient to Activate the DNA Damage Checkpoint in the Absence of Damage. Mol Cell. 2008; 10.1016/j.molcel.2008.03.023 18471973 PMC2879338

[28] Bensimon A, Aebersold R, Shiloh Y. Beyond ATM: The protein kinase landscape of the DNA damage response. FEBS Letters. 2011; 10.1016/j.febslet.2011.05.013 21570395

[29] Greer YE, Gao B, Yang Y, Nussenzweig A, Rubin JS. Lack of casein kinase 1 delta promotes genomic instability—The accumulation of DNA damage and down-regulation of checkpoint kinase 1. PLoS ONE. 2017; 10.1371/journal.pone.0170903 28125685 PMC5268481

[30] Gregory MA, Qi Y, Hann SR. Phosphorylation by Glycogen Synthase Kinase-3 Controls c-Myc Proteolysis and Subnuclear Localization. J Biol Chem. 2003; 10.1074/jbc.M310722200 14563837

[31] Welcker M, Orian A, Jin J, Grim JA, Harper JW, Eisenman RN, et al The Fbw7 tumor suppressor regulates glycogen synthase kinase 3 phosphorylation-dependent c-Myc protein degradation. Proc Natl Acad Sci. 2004; 10.1073/pnas.0402770101 15150404 PMC428477

[32] Reinhardt HC, Yaffe MB. Kinases that control the cell cycle in response to DNA damage: Chk1, Chk2, and MK2. Current Opinion in Cell Biology. 2009; 10.1016/j.ceb.2009.01.018 19230643 PMC2699687

[33] Picco V, Pagès G. Linking JNK Activity to the DNA Damage Response. Genes and Cancer. 2013; 10.1177/1947601913486347 24349633 PMC3863338

[34] Wu ZH, Shi Y, Tibbetts RS, Miyamoto S. Molecular linkage between the kinase ATM and NF-κB signaling in response to genotoxic stimuli. Science. 2006; 10.1126/science.1121513 16497931

[35] Bulavin D V., Higashimoto Y, Popoff IJ, Gaarde WA, Basrur V, Potapova O.Initiation of a G2/M checkpoint after ultraviolet radiation requires p38 kinase. Nature. 2001; 10.1038/35075107 11333986

[36] Guo Z, Kozlov S, Lavin MF, Person MD, Paull TT. ATM activation by oxidative stress. Science. 2010; 10.1126/science.1192912 20966255

[37] Paull TT. Mechanisms of ATM Activation. Annu Rev Biochem. 2015; 10.1146/annurev-biochem-060614-034335 25580527

[38] Hilioti Z, Gallagher DA, Low-Nam ST, Ramaswamy P, Gajer P, Kingsbury TJ. GSK-3 kinases enhance calcineurin signaling by phosphorylation of RCNs. Genes Dev. 2004; 10.1101/gad.1159204 14701880 PMC314273

[39] Brazill DT, Thorner J, Martin GS. Mck1, a member of the glycogen synthase kinase 3 family of protein kinases, is a negative regulator of pyruvate kinase in the yeast Saccharomyces cerevisiae. J Bacteriol. 1997; 10.1128/jb.179.13.4415-4418.1997 PMC179270 9209064

[40] Griffioen G, Swinnen S, Thevelein JM. Feedback inhibition on cell wall integrity signaling by Zds1 involves Gsk3 phosphorylation of a cAMP-dependent protein kinase regulatory subunit. J Biol Chem. 2003; 10.1074/jbc.M210691200 12704202

[41] Neigeborn L, Mitchell AP. The yeast MCK1 gene encodes a protein kinase homolog that activates early meiotic gene expression. Genes Dev. 1991; 10.1101/gad.5.4.533 2010083

[42] Schwartz MA, Madhani HD. Principles of MAP Kinase Signaling Specificity in *Saccharomyces cerevisiae*. Annu Rev Genet. 2004; 10.1146/annurev.genet.39.073003.112634 15568991

