# Correction: Mck1 kinase is a new player in the DNA damage checkpoint pathway

**DOI:** 10.1371/journal.pgen.1008710

**Published:** 2020-03-19

**Authors:** The PLOS Genetics Staff

## Notice of republication

This article was republished on November 8, 2019, to correct a linking error introduced during the typesetting process. The publisher apologizes for the error. Please download this article again to view the correct version.
